# A New Methodology for Quantification of Alternatively Spliced Exons Reveals a Highly Tissue-Specific Expression Pattern of WNK1 Isoforms

**DOI:** 10.1371/journal.pone.0037751

**Published:** 2012-05-31

**Authors:** Emmanuelle Vidal-Petiot, Lydie Cheval, Julie Faugeroux, Thierry Malard, Alain Doucet, Xavier Jeunemaitre, Juliette Hadchouel

**Affiliations:** 1 INSERM UMR970 - Paris Cardiovascular Research Center - Paris, France; 2 University Paris-Descartes, Sorbonne Paris Cité, Faculty of Medicine, Paris, France; 3 UPMC Univ Paris 06 and INSERM UMRS 872 and CNRS ERL726 - Cordeliers Research Center - Paris, France; 4 CNRS, LCTS, UMR5801, Pessac, France; 5 AP-HP, Department of Genetics, Hôpital Européen Georges Pompidou, Paris, France; University of Geneva, Switzerland

## Abstract

Mutations in the *WNK1* gene, encoding a serine-threonine kinase of the WNK (With No lysine (K)) family, have been implicated in two rare human diseases, Familial Hyperkalemic Hypertension (FHHt) and Hereditary Sensory and Autonomic Neuropathy type 2 (HSAN2). Alternative promoters give rise to a ubiquitous isoform, L-WNK1, and a kidney-specific isoform, KS-WNK1. Several other isoforms are generated through alternative splicing of exons 9, 11 and 12 but their precise tissue distribution is not known. Two additional exons, 8b and HSN2, involved in HSAN2, are thought to be specifically expressed in the nervous system. The purpose of this study was to establish an exhaustive description of all WNK1 isoforms and to quantify their relative level of expression in a panel of human and mouse tissues and in mouse nephron segments. For the latter purpose, we developed a new methodology allowing the determination of the proportions of the different isoforms generated by alternative splicing. Our results evidenced a striking tissue-specific distribution of the different isoforms and the unexpected presence of exon HSN2 in many tissues other than the nervous system. We also found exon 26 to be alternatively spliced in human and identified two new exons, 26a and 26b, within intron 26, specifically expressed in nervous tissues both in humans and mice. *WNK1* should therefore no longer be designated as a 28- but as a 32-exon gene, with 8 of them - 8b, HSN2, 9, 11, 12, 26, 26a and 26b - alternatively spliced in a tissue-specific manner. These tissue-specific isoforms must be considered when studying the different roles of this ubiquitous kinase.

## Introduction

WNK1 (With No lysine (K) 1) is a serine threonine kinase of the WNK family, which comprises 4 members (WNK1 to WNK4). Large deletions of the first intron of the *WNK1* gene are implicated in Familial Hyperkaliemic Hypertension (FHHt), a rare autosomal dominant disease characterized by a dysfunction of the distal nephron [1]. The majority of studies conducted to date have focused on its role in the kidney [2]. However, WNK1 is ubiquitous and recent studies have started to highlight its functions in extrarenal tissues (for review, see [3]). WNK1 plays a major role in cardiovascular tissues both in the adult mouse, where it regulates vasoconstriction in response to α_1_-adrenergic receptors [4], and in the mouse embryo, where it participates in arterio-venous specification [5], [6]. In addition, *in vitro* studies clearly implicate WNK1 in cell migration, division and differentiation [7], [8], possibly through the MAP kinase pathway [8], [9], and large scale sequencing studies have shown a link between somatic mutations of *WNK1* and several types of cancer [10], [11], [12]. Finally, mutations in exon HSN2 of *WNK1*, located within intron 8, are responsible for a form of congenital insensitivity to pain, HSAN2 (Hereditary Sensory and Autonomic Neuropathy type 2) [13]. Exon HSN2 is described as being exclusively expressed in the nervous system, associated with another exon named 8b in some transcripts [13], but the role of the protein sequence encoded by 8b and HSN2 is unknown.

WNK1 is encoded by a very large gene located on human chromosome 12, which spans over 160 kb and has so far been considered as containing 28 exons. It gives rise to multiple isoforms that may explain the different roles of the kinase. Alternative promoters control the expression of L- and KS-WNK1 [14], [15], which stand for Long and Kidney-Specific WNK1 respectively. L-WNK1, with its transcript starting at exon 1, is ubiquitously expressed under the control of proximal promoters [14]. KS-WNK1 is a shorter kinase-deficient isoform as its transcription starts at exon 4a, replacing the first 4 exons which encode most of the kinase domain. It is highly and specifically expressed in the Distal Convoluted Tubule (DCT) and Connecting Tubule (CNT) of the nephron. The situation is much more complex than this as *WNK1* is also subjected to alternative splicing [3]. Exons 11 and 12 are alternatively spliced in human and mouse as well as exon 9 in human only [14], [15], [16], but the pattern of expression of the corresponding transcripts has not been examined in detail.

In this study, we first sought to identify all alternatively spliced exons of *WNK1*. We confirmed the splicing of exons 9, 11, 12, 8b and HSN2 and identified exon 26 and two new exons, 26a and 26b, as novel alternatively spliced exons of *WNK1*. We then established the pattern of expression of these 8 alternatively spliced exons in a panel of human and mouse tissues and developed a new methodology to quantify the proportion of each WNK1 isoform in each tissue of interest. We thus show that the different isoforms display a striking tissue-specificity, which is partially conserved between human and mouse. When investigating the multiple roles of WNK1 in the different tissues, it now appears that one must take into account the tissue-specific isoforms of the kinase.

## Results

### A new procedure for the quantification of WNK1 isoforms

The aim of our study was not only to define the pattern of expression of all WNK1 transcripts generated by alternative splicing but also to estimate precisely how much of each isoform is present in a given tissue. Relative quantification with Real Time Quantitative RT-PCR (RT-QPCR) using the classical 2^−ΔΔCT^ method allows the determination of the pattern of expression of a given gene in a panel of tissues or the comparison of its level of expression between two conditions, with one tissue or condition used as a calibrator [17]. However, it does not allow the comparison of the level of expression of two transcripts in a given tissue or condition, in our case two WNK1 isoforms. In order to circumvent this limitation, we developed a new analytical procedure, described in detail in the Methods section, which allowed us to determine the relative proportions of each WNK1 isoform in a panel of human and mouse tissues using RT-QPCR. Briefly, we used a “never-spliced” exon of WNK1 as a reference, instead of a classical housekeeping gene. Then, following the simple principle that the sum of all WNK1 isoforms in a tissue equals the level of expression of a ‘never-spliced’ WNK1 exon, in our case exon 8, we wrote equations in order to determine the real proportions of WNK1 isoforms. The values obtained for all WNK1 isoforms in both human and mouse are given in the subsequent figures and Tables S1, S2, S3.

### Pattern of expression of L-and KS-WNK1 in human and mouse

**Figure 1:**
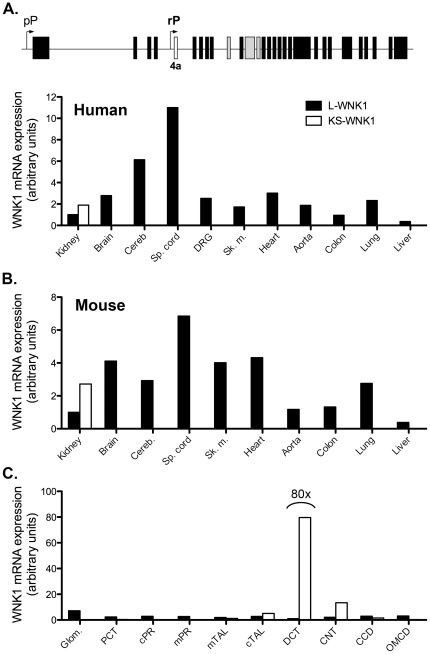
Level of expression of L-WNK1 and KS-WNK1 mRNA in a panel of human and mouse tissues. (A) Upper panel: Schematic representation of the *WNK1* gene. The proximal promoter (pP) drives the transcription of L-WNK1. The renal promoter (rP) controls the expression of KS-WNK1, whose transcription starts at exon 4a (white box), specific to this isoform. Expression of L-WNK1 was quantified by RT-QPCR using primers recognizing exons 2–3 in humans (lower panel in A) and 1–2 in mice (B). Expression of KS-WNK1 was quantified by RT-QPCR using primers recognizing exons 4a-5. *ubc* was used as a reference gene. The expression level of L-WNK1 was arbitrarily set to 1 in the kidney. Panel C presents the data obtained in microdissected mouse nephron segments using *RPL26* as a reference gene. The expression level of L-WNK1 was arbitrarily set to 1 in the DCT. Glom: glomerulus -PCT: Proximal Convoluted Tubule - cPR:cortical Pars Recta – mPR:medullary Pars Recta – mTAL: medullary Thick Ascending Limb of Henle's loop – cTAL: cortical Thick Ascending Limb of Henle's loop – DCT: Distal Convoluted Tubule – CNT: Connecting tubule – CCD: cortical Collecting Duct – OMCD: Outer Medullary Collecting Duct. Expression of L-WNK1 is arbitrarily set to 1 in the kidney (B) and in the DCT (C) and expression of KS-WNK1 is expressed relatively to L-WNK1, the ratio of KS-WNK1 over L-WNK1 being obtained as described in the method section.

Most data about the pattern of expression of WNK1 come from northern blot analyses performed with commercial multi-tissues blots, including those identifying the ubiquitous distribution of L-WNK1 and the kidney specific expression of KS-WNK1 [14], [15], [16], [18]. We further characterized the tissue distribution of these two isoforms by quantifying their expression using RT-QPCR on a large panel of human and mouse tissues (Figure 1A and B). Surprisingly, in both species, the strongest expression of L-WNK1 is seen in the spinal cord. In brain, cerebellum, dorsal root ganglia (DRG), skeletal muscle, heart, aorta and lung, the expression is fairly strong in both species. The expression of L-WNK1 is weaker in the kidney and diminishes further in the colon. In both species, the lowest expression of L-WNK1 is seen in the liver. Our results confirm that the expression of KS-WNK1 is restricted to the kidney.

Expression of L-and KS-WNK1 was further characterized within the mouse kidney, in microdissected nephron segments (Figure 1C and Table S3). RT-QPCR confirmed that L-WNK1 is expressed all along the nephron at similar levels, except for a higher expression in the glomerulus and a lower expression in the DCT. In contrast, our analysis revealed that KS-WNK1 expression pattern is broader than previously reported. Its main sites of expression are the DCT and CNT. In the DCT, KS-WNK1 represents 99% of WNK1 isoforms and is expressed 80 times more than L-WNK1. In the CNT, KS-WNK1 represents 64% of WNK1 isoforms, respectively, but its expression level is 6 times less than in the DCT. KS-WNK1 is also the predominant isoform in the cTAL (64% of WNK1 isoforms), even though it is expressed 16 times less than in the DCT. Finally, KS-WNK1 is expressed at a very low level in the mTAL and CCD (50 to 65 times less than in the DCT), where it represents between 35 and 40% of WNK1 isoforms. In order to rule out that the low expression seen in the segments adjacent to the DCT is due to a contamination of these segments preparations by DCTs, we performed a RT-QPCR on the same preparations with primers recognizing the DCT-specific NaCl co-transporter NCC. As expected, NCC transcripts were detected only in the DCT preparations (Figure S1), thus showing that cDNAs from DCT do not contaminate other segments.

### Identification of three alternative splicing regions in *WNK1*

**Figure 2:**
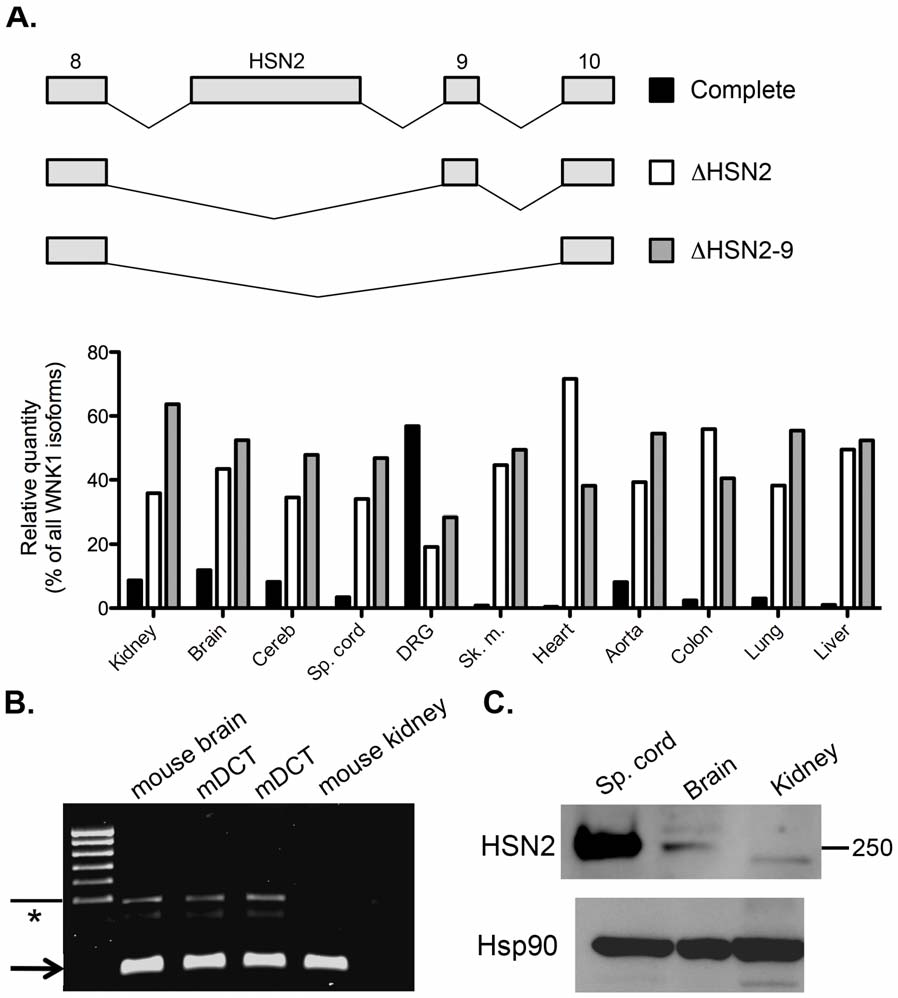
Pattern of expression and relative proportions of the human splice variants of region 8–10. (A) Upper panel: schematic representation of the different splicing events that can occur in this region. Lower panel: relative quantities of these variants in a panel of human tissues, expressed as a percentage of total WNK1 expression. Cereb.: Cerebellum; Sp. cord: spinal cord; DRG: Dorsal Root Ganglia; Sk. m.: Skeletal muscle. (B) A HSN2-containing WNK1 transcript is expressed in mDCT cells. RT-QPCR was performed between exon 8 and HSN2, resulting in the amplification of a 228 bp fragment (arrow), corresponding to the “Δ8b” isoform, and a 486 bp fragment (arrowhead), corresponding to the complete isoform. RNA extracts from mouse brain, expressing both isoforms, and from mouse kidney, expressing mostly the “Δ8b” isoform, were used as control. The weak band indicated by an asterisk results from a non-specific amplification (verified by sequencing). (C) The HSN2-containing protein is detected in the mouse kidney. Immunoblot of various mouse tissues incubated with an antibody directed against the portion of the protein encoded by HSN2. A strong signal is detected in the spinal cord and a lower signal in the brain and renal cortex. An immunoblot incubated with a HSP90 antibody shows equal loading of the different samples.

**Figure 3:**
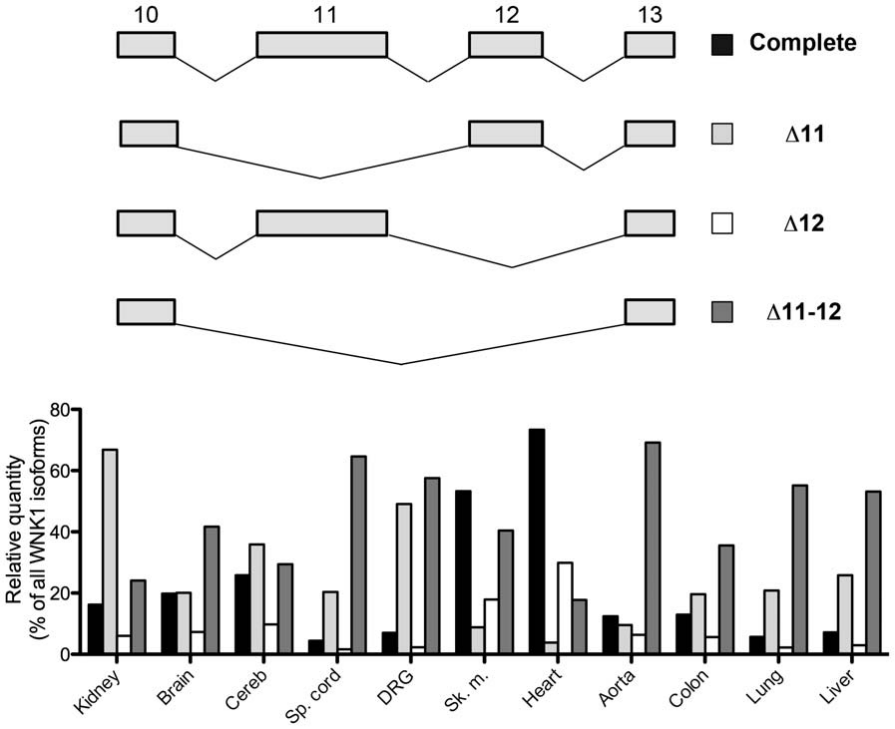
Pattern of expression and relative proportions of the human splice variants of region 10–13. Upper panel: schematic representation of the different splicing events that can occur in this region. Lower panel: relative quantities of these variants in a panel of human tissues, expressed as a percentage of total WNK1 expression.

**Figure 4:**
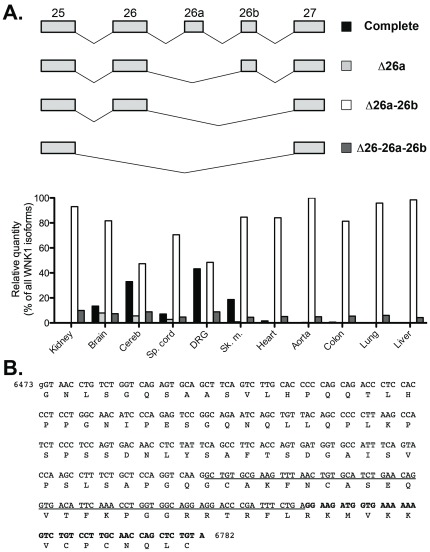
Pattern of expression and relative proportions of the human splice variants of region 25–27. (A) Upper panel: schematic representation of the different splicing events that can occur in this region. Lower panel: relative quantities of these variants in a panel of human tissues, expressed as a percentage of total WNK1 expression. (B) DNA and amino acid sequences of exons 26, 26a and 26b. Sequences of exon 26a and 26b are respectively underlined and in bold.

In order to identify regions likely to be alternatively spliced, we combined three screening methods. First, we reviewed all relevant articles in the MEDLINE database; second, we screened the EST database for transcripts resulting from alternative splicing and, third, we amplified overlapping regions of WNK1 mRNA by RT-PCR. This allowed us to determine three regions of interest. The first is located between exons 8 and 10 and contains three alternative exons - 8b, HSN2, and 9 (Figure 2). The second, located between exons 10 and 13, contains two alternative exons - 11 and 12 (Figure 3). The third region, located between exons 25 and 27, has never been studied before. Our results show that exon 26 is alternatively spliced and identify two novel alternative exons, 26a and 26b (Figure 4).

### Exons 8b, HSN2 and 9

Three exons are submitted to alternative splicing in the region located between exons 8 and 10. Exon 9 is spliced out of roughly half of the transcripts in all human tissues (Figure 2A). This splicing event does not occur in mice. Transcripts containing the remaining two, exons 8b and HSN2, were previously cloned and sequenced in mouse tissues [13], where they were described as specifically expressed in the nervous system. Our study confirms that the highest expression of HSN2 is observed in the mouse dorsal root ganglia (DRG) and spinal cord, where it is present in 57% of WNK1 isoforms (Figure S1). However, its expression is not restricted to the nervous system. RT-PCR performed on the mDCT cell line, isolated from mouse DCT [19], showed that HSN2 expression outside the nervous system cannot be attributed solely to tissue innervations (Figure 2B). The HSN2-containing WNK1 protein, WNK1/HSN2, is also detected in the mouse kidney (Figure 2C). KS-WNK1 appears to contain exon HSN2 as well, as a PCR product is amplified between exons 4a and HSN2 (data not shown). This is further confirmed by quantification of the alternative exons in the different nephron segments, which shows that the expression of HSN2, even though very weak, follows that of the combination of L- and KS-WNK1, with a low level of expression along the kidney and a higher expression in the DCT (Figure S2). We show that exon 8b is only included when HSN2 is present and that its expression in mice is predominantly detected in the nervous system; however 12% of WNK1 isoforms in the aorta also contain this exon, a level comparable to that of the cerebellum.

In human, HSN2-containing transcripts are also expressed ubiquitously, with a much higher level of expression in the dorsal root ganglia, where 57% of WNK1 transcripts contain this exon (Figure 2A). WNK1/HSN2 represent only 3% of WNK1 isoforms in the spinal cord in human, thus demonstrating that the very strong expression of L-WNK1 in this tissue (Figure 1) is not due to the expression of this isoform. Exon 9 is always present in the WNK1/HSN2 isoform. Even though a genomic sequence highly homologous to that of mouse exon 8b can be found in the human genome database, we could not detect expression of this exon in human tissues, either by RT-PCR or RT-QPCR.

### Exons 11 and 12

In both humans and mice, exons 11 and 12 are subjected to alternative splicing. Exon 11 can be spliced alone (“Δ11” isoform) or together with exon 12 (“Δ11–12” isoform) but exon 12 (“Δ12” isoform) is rarely spliced alone (Figure 3). Indeed, the “Δ12” isoform is expressed at very low levels in all tissues examined, except in the heart in both species and the skeletal muscle in human.

Each isoform shows a very specific pattern of expression (Figure 3 and Figure S3). The isoform including both exons (“complete” isoform) is expressed only in the heart, the skeletal muscle and neural tissues in both species but the level of expression differs between species. A very high expression of this isoform is observed in the human heart, where it represents 73% of all WNK1 isoforms, while it represents 30–48% of WNK1 isoforms in every aforementioned tissue in mice. “Δ11–12” is the predominant isoform in the aorta, particularly in human (69% and 52% of all WNK1 isoforms in human and mouse respectively).

The major isoform expressed in the kidney is “Δ11”, which represents ∼70% of WNK1 renal transcripts in both humans and mice. Exon 12 is the most abundant alternatively spliced exon in the nephron (Figure S3C). In order to determine if this exon is included in L-WNK1 and/or KS-WNK1, we calculated the ratio of exon 12 over exon 8 to estimate the proportion of transcripts containing this exon in each nephron segment (Figure S3D). This ratio is similar in all nephron segments (∼80%), whether they express L-WNK1 only, such as the proximal tubules, or a large majority of KS-WNK1, such as the DCT (Figure S3D). This indicates that (1) 80% of L-WNK1 transcripts contain exon 12 in the segments expressing only L-WNK1 and (2) that 80% of KS-WNK1 transcripts contain exon 12 in the segments where KS-WNK1 is predominant. However, we cannot draw a conclusion about L-WNK1 transcripts in those segments as L-WNK1 is expressed at a very low level compared to KS-WNK1, particularly in the DCT where L-WNK1 represents only 1.5% of all WNK1 transcripts.

### Exons 26, 26a and 26b

We identified for the first time the region between exons 25 and 27 as a region of interest concerning alternative splicing (Figure 4 and Figure S4). The amplification of WNK1 mRNA between exons 25 and 27 revealed, as expected, a band corresponding to the full length transcript (exons 25–26–27). The rare splicing of exon 26 could also be detected, but only in human. In neural tissues and skeletal muscle, we also detected PCR products longer than expected. Sequencing of these products revealed the existence of two new exons within intron 26 of *WNK1*, which we named 26a and 26b. While HSN2 does not appear to be specific to the nervous system, exons 26a and 26b are exclusively expressed in neural tissues and skeletal muscle. In both species, 26b can be present alone, especially in the central nervous system, whereas 26a is only present together with 26b. Exons 26a and 26b, 100% conserved between mouse and human, are respectively 72 and 42 nucleotides long. Their presence does not induce the appearance of a frameshift or a stop codon.

## Discussion

Careful examination of the different isoforms of WNK1 revealed a much more complex situation than initially suspected [3]. Of the 28 usually described exons of WNK1 [16], four in humans (9, 11, 12 and 26) and two in mice (11 and 12) are subjected to alternative splicing. Because of its later discovery, exon 4a was not initially included in the 28 exons of WNK1. WNK1 contains four other exons: exons 8b and HSN2, discovered in the nervous system [13], and exons 26a and 26b, described here for the first time. Our study not only revealed the complex pattern of WNK1 isoforms but also allowed the quantification of each isoform in a panel of human and mouse tissues.

Relative quantification with RT-QPCR, based on the 2^−ΔΔCT^ formula or the efficiency-corrected formula [20], is the most commonly used method to establish the pattern of expression of a given gene in different tissues or experimental conditions, with one tissue or condition used as a calibrator [17]. However, our goal was not only to establish the pattern of expression of WNK1 isoforms, but also to quantify the proportions of these isoforms in each tissue of interest. We developed a new methodology to analyze data from RT-QPCR in the case of multiple isoforms generated from one gene. This technique is a simple and inexpensive tool to quantify alternative splicing. Noteworthy, this methodology can also be extended to compare the expression of different genes as long as they share a 100% homologous region which can be used as a reference gene. This will often be the case for homologous genes.

The inclusion or deletion of any combination of the WNK1 alternative exons does not generate a frameshift or a stop codon. WNK1 is a large 250 kDa protein and the prediction of the potential role of these exons is difficult in the absence of a three-dimensional model of the WNK1 protein structure, as only the crystal structure of the kinase domain has been determined [21]. Computational tools do not predict motifs in these exons, which does not exclude their potentially important role in protein structure/function. This is obvious in the case of HSN2, as its mutated form leads to loss of pain perception, even though no functional motif can be identified.

An unexpected result was the clear amplification of a HSN2-containing transcript by PCR in non-neuronal tissues. Why this band was not detected previously [13] is not clear. While the authors did report a band in the kidney, they attributed this to contamination by adrenal tissue. In our case, kidney dissection was clear of adrenal tissue and the detection of HSN2 could not be attributed to the innervations of the kidney since we were able to amplify the same HSN2 transcript in different renal epithelial cell lines. These results do not diminish the crucial role of WNK1/HSN2 in the nervous system but opens the possibility that this isoform might have a function outside the nervous system. Interestingly, we observed a particularly high level expression of HSN2-containing transcripts in the human dorsal root ganglia, confirming the study conducted in mice [13].

One limitation of our study is that we considered all the studied tissues as a whole, the only exception being the kidney. All these tissues contain different cell types, which may express different WNK1 isoforms. This means that an isoform which is highly expressed in only one cell type could appear as a minor isoform in the whole tissue. For instance, there is only a two-fold difference between KS-WNK1 and L-WNK1 in the whole kidney, whereas KS-WNK1 is 80-times more expressed than L-WNK1 in DCT cells. Since WNK1 function has been mostly studied in the kidney, we focused on this tissue but the same could be true in others. HSN2-containing transcripts in the dorsal root ganglia may represent 100% of all transcripts in the satellite cells that envelop sensory neurons [13]. Similarly, the strong expression observed in the cerebellum probably results from an especially high expression in the granular layer and Purkinje cells [22], with fairly weak expression elsewhere.

These complex and tissue-specific alternative splicing events must be taken into account when interpreting some of the previously published data. For instance, alternative splicing of exon 12 is of particular importance as the main study which localized WNK1 by immunohistochemistry used an antibody against the portion of the protein encoded by exon 12 [23]. More importantly, due to the incomplete knowledge of the tissue-specific isoforms of WNK1, none of the numerous studies conducted in *Xenopus oocytes* or cell lines has ever discussed which transcript was used and the transcript has never been adapted to the organ studied. Most *in vitro* studies on the function of WNK1 were performed with the rat isoform cloned by the laboratory of M. Cobb [18], which happens to be the “Δ11–12” isoform. To our knowledge, no renal study has been conducted with the isoform expressed specifically in this organ, i.e. the “Δ11” isoform. Such a detailed analysis has recently been reported for *WNK3*, the alternative splicing of which generates four different transcripts, with some being specifically expressed in the nervous system. If a first study concluded that brain and kidney WNK3 isoforms have opposite effects on NCC [24], a more recent and exhaustive study reported that all WNK3 transcripts display similar activities toward the co-transporters of the SLC12 family [25].

In conclusion, *WNK1* is a complex 32 exon-gene with multiple tissue-specific isoforms. Exons 8b, HSN2, 26a and 26b are newly identified alternatively spliced exons that may play a crucial role, as has already been demonstrated for HSN2. To be exhaustive, one should also add exon 4a, discovered a few years after the kinase was first cloned, making the total exon number 33. Of the classically described 28 exons of the gene, 4 are also alternatively spliced, namely exons 9, 11, 12 and 26. In total, 9 WNK1 exons are alternatively spliced, some expressed in a tissue-specific manner like exon 4a in the kidney and exons 26a and 26b in the nervous system, some expressed in all tissues investigated, with different preferential expressions like exon 11 in the heart and exon HSN2 in the neural tissues, and some expressed ubiquitously with no preferential expression, such as exons 9 and 26. The complexity of this gene must be taken into account in future studies of both the renal and extra-renal roles of WNK1.

## Materials and Methods

### Total RNA from mouse and human tissues

All mouse studies were performed in accordance with the French government animal policy (agreement number 75–1254). Total RNA was extracted from mouse tissues (4 C57Bl/6N 3 month-old males) using the Nucleospin® RNA II extraction kit (Macherey-Nagel) according to the manufacturer's instructions. Human total RNA extracts from various tissues were purchased from BD Clontech.

### RT-PCR and DNA sequence analysis

Total RNA was treated with DNase (DnaseI, Ambion) and reverse transcribed using Superscript II reverse transcriptase and Random Primers (Life Technologies™). Alternative splicing was explored with primers complementary to non-spliced exons flanking the region of interest. The sequence of all primers used in this study is given in Table S4. The amplified fragments were visualized by electrophoresis on agarose gels. PCR products differing from the expected size were purified and subcloned into pGEM®-T (Promega) and sequenced using the Sanger method (GATC Biotech). The sequence of the complete human WNK1 coding sequence, containing exons HSN2, 26a and 26b, was deposited in Genbank (Accession number: JQ358908).

### Real-time quantitative RT-PCR

Real-time quantitative RT-PCR (RT-QPCR) was carried out using intercalation of SYBR green (qPCR MasterMix Plus for SYBR®; Eurogentec) on a Chromo4 continuous fluorescence detector (MJ Research, Bio-Rad laboratories, Waltham, MA). Human or mouse cDNA was serially diluted by 1/5 to generate standard curves. Primers were used only if the efficiency calculated from the standard curves was 2±0.05 and if the melting curve showed a single peak. No amplification was detected in samples that did not undergo reverse transcription.

Two reference genes, *18S* and *GAPDH* for human tissue, *18s* and *ubiquitin C* (*ubc*) for mouse tissue, and *ubc* and *RPL26* for mouse nephron segments were tested. Both reference genes gave similar results and the reference gene with the most stable expression (*18S* for human, *ubc* for mouse tissues and *RPL26* for nephron segments) was chosen to establish the pattern of expression of L- and KS-WNK1 in the tissue panel or nephron segments, using the 2^−ΔΔCT^ formula with the kidney or the DCT arbitrarily fixed as a calibrator. L-WNK1 expression was quantified using primers recognizing exons 1 and 2 (mouse) or 2 and 3 (human). KS-WNK1 expression was quantified using primers recognizing exons 4a (specific of KS-WNK1, Figure 1A) and 5. To examine WNK1 isoforms generated by alternative splicing, we designed primer pairs to overlap exons that are contiguous only in the isoform of interest (Figure S5).

### Determination of the relative quantities of WNK1 isoforms in a given tissue

As described in the Results section, the relative quantification using the 2^−ΔCT^ formula [17] does not indicate the real ratio of the gene of interest over the reference gene. For each couple of primers, the correction factor that would give the real value of this ratio is unknown and therefore the relative quantity of two transcripts in a given sample cannot be determined. We circumvented this limitation by developing a methodology to determine the correction factor which, applied to the 2^−ΔCT^ ratio, gives the real ratio of the level of expression of the gene of interest over the reference gene.

The conditions needed to apply our method are the following:

1. a portion of the mRNA that is common to all isoforms must be determined and used as a internal reference gene.
2. primers should be designed to recognize specifically mutually exclusive isoforms (as illustrated in the case of region 10–13 in Figure S5).
3. The RT-QPCR should be run in at least as many tissues or conditions as there are different isoforms.

The following equations can then be written in every sample:
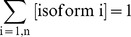


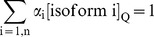
where [isoform i] is the “true” expression ratio of isoform i relative to the reference gene, and 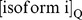 is the ratio given by the 2^−ΔCT^ formula, where ΔCT is the difference in threshold cycles for isoform i and the reference gene (CT_i_−CT_ref_) (see detailed formula below), which must be corrected by a factor 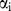 that is *a priori* unknown. 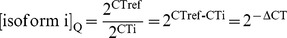
The n correction factors are determined using a simple algorithm run in C++ that calculates the average and the standard deviation of the sum 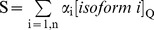 in all analysed tissues for all possible combinations of α_i_, with a minimal increment of 0.1 from 0.1 to 10. A filter is applied to the results stored in a MySQL database to retrieve the combination of correction factors (α_1_ to α_n_) that yields a value of S between 0.98 and 1.02 in the ≥n tissues, with the lowest relative standard deviation. This combination is then used to determine the true fraction of each isoform in each tissue.

Noteworthy, the correction factors determined by the equations include adjustment for the length of the amplicon, so there is no need to take that factor into account [26] when using our method. Our methodology applied to the results obtained using a formula that included the efficiency of the PCR reactions [20], [26], or using values derived from a standard curve yielded the same results, which is not surprising since the primers were only used if the corresponding efficiency was very close to 2, a condition necessary for the validity of the 2^−ΔΔCT^ formula [17]. Results obtained for all WNK1 isoforms in both human and mouse are given in Tables S1, S2, S3. The unknown correction factors yield a sum of approximately 1 (or 100%) in each of the examined tissues, which validates our approach. The intrinsic imprecision of RT-QPCR, mainly due to pipetting inaccuracy, explains the fact that the sum of isoforms in each tissue is not exactly equal to 100%. Indeed, the same RT-QPCR experiment performed several times always gives slightly different results, with a precision of about 10% in our hands, explaining why the standard deviation of the means of the sums is around 5–10% for each isoform, and is expected to be higher when more isoforms are examined.

#### Example of L-WNK1 and KS-WNK1

Assuming that the sum of the “true” KS-WNK1-to-exon 8 expression ratio (denoted by [KS-WNK1]) and the “true” L-WNK1-to-exon 8 expression ratio (denoted by [L-WNK1]) is equal to 1 (or 100%), we have in every tissue:
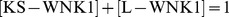
As described above, the ratios obtained by RT-QPCR (denoted 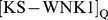 and 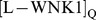) must be pondered by correction factors, such that:
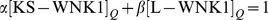
where α and β are *a priori* unknown. These factors were determined as described above. The original and corrected values are given in Table S3.

### RT-QPCR on microdissected mouse nephron segments

Glomeruli and nephron segments from 6 C57Bl/6 adult males were characterized and microdissected from liberase-treated kidneys under a binocular microscope as previously described [27], [28]. After microdissection, pools of tubules of identical structures from a single mouse were thoroughly rinsed and total RNA was extracted using the RNeasy® Micro Kit (Qiagen). Reverse transcription was performed simultaneously for all samples using the First-strand cDNA Synthesis kit for RT-PCR (Roche Diagnostics) and Real-time QPCR was performed on a LightCycler (Roche Diagnostics) with the LightCycler FastStart DNA Master SYBR Green 1 kit (Roche Diagnostics) as previously described [28].

### Immunoblotting

Preparation of renal cortex, brain and spinal cord total protein extracts from mouse tissues and immunoblotting was performed as previously described [29]. The antiWNK1/HSN2 antibody was a gift of G. Rouleau (Montreal, Canada).

## Supporting Information

Figure S1 **Verification of the absence of contamination of the microdissected segments preparations by the DCT.** The expression of the DCT-specific NaCl co-transporter NCC was quantified in the different nephron segments by RT-QPCR, using *RPL26* as a reference gene. Datas are means ± s.e.m from 6 mice. The expression level was arbitrarily fixed set to 100 in the DCT. Glom: glomerulus -PCT: Proximal Convoluted Tubule - cPR:cortical Pars Recta – mPR: medullary Pars Recta – mTAL: medullary Thick Ascending Limb of Henle's loop – cTAL: cortical Thick Ascending Limb of Henle's loop – DCT: Distal Convoluted Tubule – CNT: Connecting tubule – CCD: cortical Collecting Duct – OMCD: Outer Medullary Collecting Duct.
(PDF)

Figure S2 **Pattern of expression and relative proportions of the splice variants of region 8–10 in mice.** (A) Schematic representation of the different splicing events that can occur in this region. (B) Relative quantities of these variants in a panel of mouse tissues, expressed as a percentage of total WNK1 expression. Cereb.: Cerebellum; Sp. cord+DRG: Spinal cord+Dorsal Root Ganglia; Sk. muscle: Skeletal muscle. Unlike the human situation, exon 9 is always included in the mouse WNK1 mRNA, which could also contain an additional exon, 8b. (C) Expression level of exons 8b, HSN2 and 11 in microdissected mouse nephron segments, relative to RPL26. Exon 11 was included to highlight the low expression of exons 8 h and HSN2, compared to exon 12, described in Figure S3C. Datas are means ± s.e.m from 6 mice.
(PDF)

Figure S3 **Pattern of expression and relative proportions of the splice variants of region 10–13 in mice.** (A) Schematic representation of the different splicing events that can occur in this region. (B) Relative quantities of these variants in a panel of mouse tissues, expressed as a percentage of total WNK1 expression. (C) Expression level of exons 11 and 12 in microdissected mouse nephron segments, relative to RPL26. Datas are means ± s.e.m from 6 mice. (D) Relative quantity of WNK1 transcripts containing exon 12 in microdissected mouse nephron segments, calculated as the ratio of exon 12 over exon 8.
(PDF)

Figure S4 **Pattern of expression and relative proportions of the splice variants of region 25–27 in mice.** Upper panel: schematic representation of the different splicing events that can occur in this region. Lower panel: relative quantities of these variants in a panel of mouse tissues, expressed as a percentage of total WNK1 expression. Unlike the human situation, exon 26 is never spliced out of the mouse WNK1 mRNA.
(PDF)

Figure S5 **Schematic representation of the localization of the primers used to determine the relative quantity of the WNK1 isoforms.** The example of region 10–13 is shown. One of the primers within one pair was designed such that it overlaps exons that are contiguous only in the isoform of interest. For example, to study the expression of the “Δ11” isoform, the forward primer recognises a sequence located in exon 10 while the reverse primer overlaps exons 10 and 12.
(PDF)

Table S1 Relative quantities of WNK1 isoforms in a panel of human tissues.
(PDF)

Table S2 Relative quantities of WNK1 isoforms in a panel of mouse tissues. * Spinal cord samples also contain the Dorsal Root Ganglia.
(PDF)

Table S3 Relative quantities of L-WNK1 and KS-WNK1 in microdissected mouse nephron segments. [L-WNK1]_Q_ and [KS-WNK1]_Q_ are the values of the ratio of each transcript over exon 8, given by the 2^−ΔCT^ formula. [L-WNK1] and [KS-WNK1] are the “true” expression ratio. α and β are the calculated correction factors. The ratios given here, being expressed over exon 8, represent the proportion of L-or KS-WNK1 amongst all WNK1 transcripts. Glom: Glomerulus - PCT: Proximal Convoluted Tubule - cPR: cortical Pars Recta - mPR: medullary Pars Recta - mTAL: medullary Thick Ascending Limb of Henle's loop - cTAL: cortical Thick Ascending Limb of Henle's loop - DCT: Distal Convoluted Tubule - CNT: Connecting tubule - CCD: cortical Collecting Duct - OMCD: Outer Medullary Collecting Duct.
(PDF)

Table S4 Sequence of all primers used in this study.
(XLS)
