# Supplementary figures and images for: A New Methodology for Quantification of Alternatively Spliced Exons Reveals a Highly Tissue-Specific Expression Pattern of WNK1 Isoforms
Source: PLoS One. 2012 May 31;7(5):e37751. doi: 10.1371/journal.pone.0037751 (PMC3365125; doi:10.1371/journal.pone.0037751)

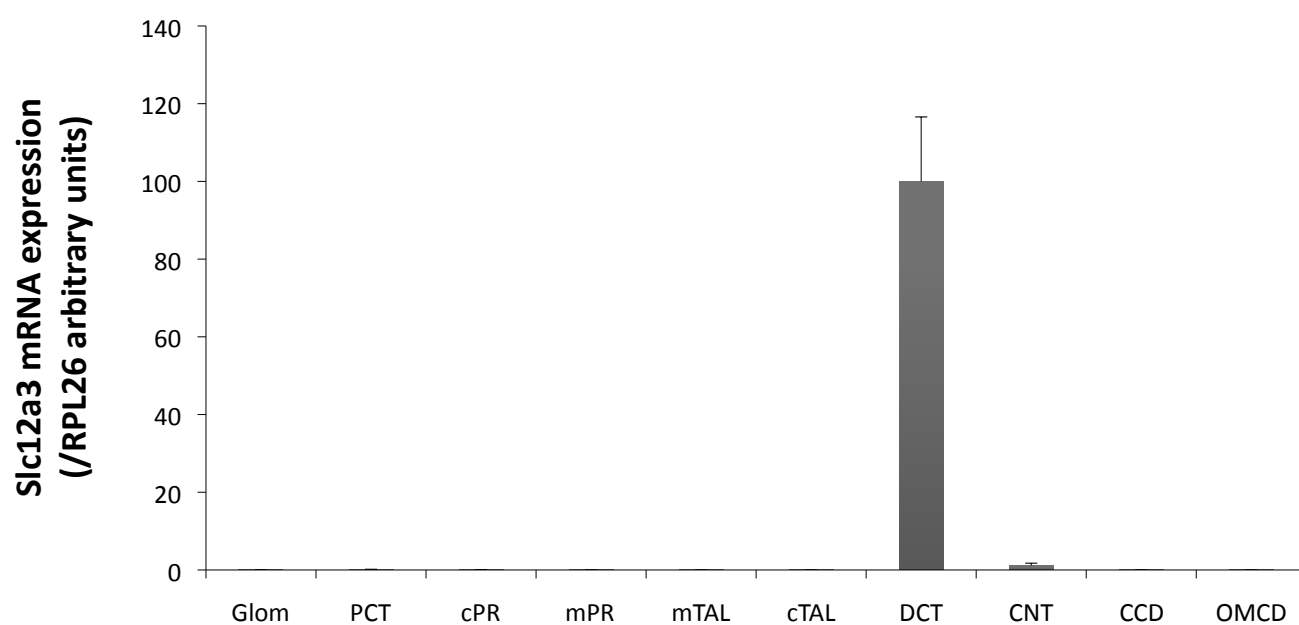

**Figure S1**

Supplement: Figure S1 — Verification of the absence of contamination of the microdissected segments preparations by the DCT. The expression of the DCT-specific NaCl co-transporter NCC was quantified in the different nephron segments by RT-QPCR, using RPL26 as a reference gene. Datas are means ± s.e.m from 6 mice. The expression level was arbitrarily fixed set to 100 in the DCT. Glom: glomerulus -PCT: Proximal Convoluted Tubule - cPR:cortical Pars Recta – mPR: medullary Pars Recta – mTAL: medullary Thick Ascending Limb of Henle's loop – cTAL: cortical Thick Ascending Limb of Henle's loop – DCT: Distal Convoluted Tubule – CNT: Connecting tubule – CCD: cortical Collecting Duct – OMCD: Outer Medullary Collecting Duct. (PDF) [file pone.0037751.s001.pdf]

A.

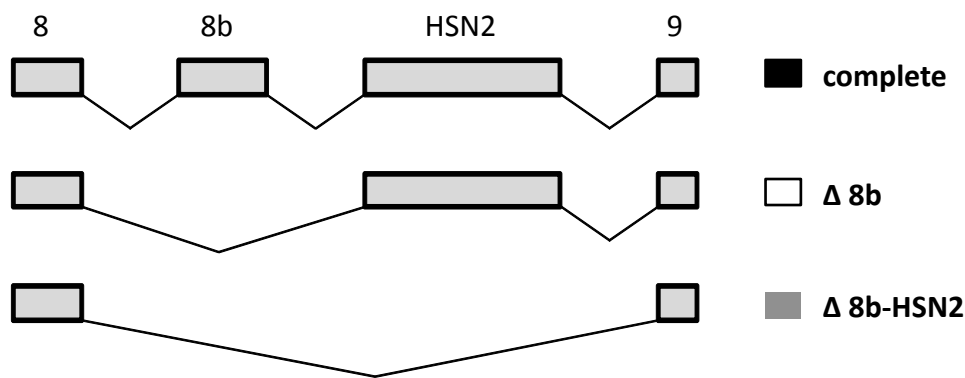

B.

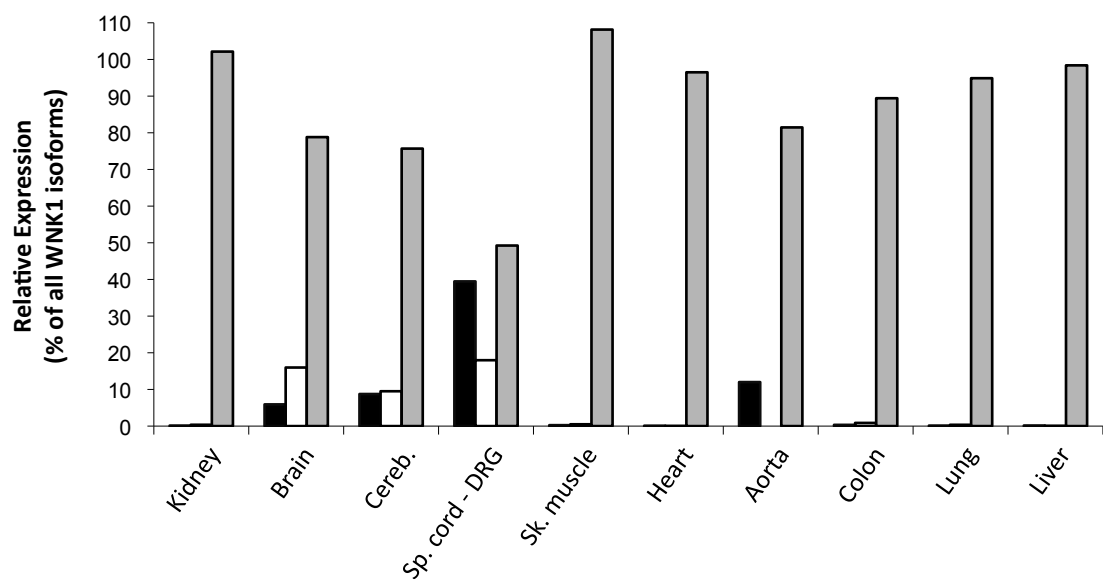

C.

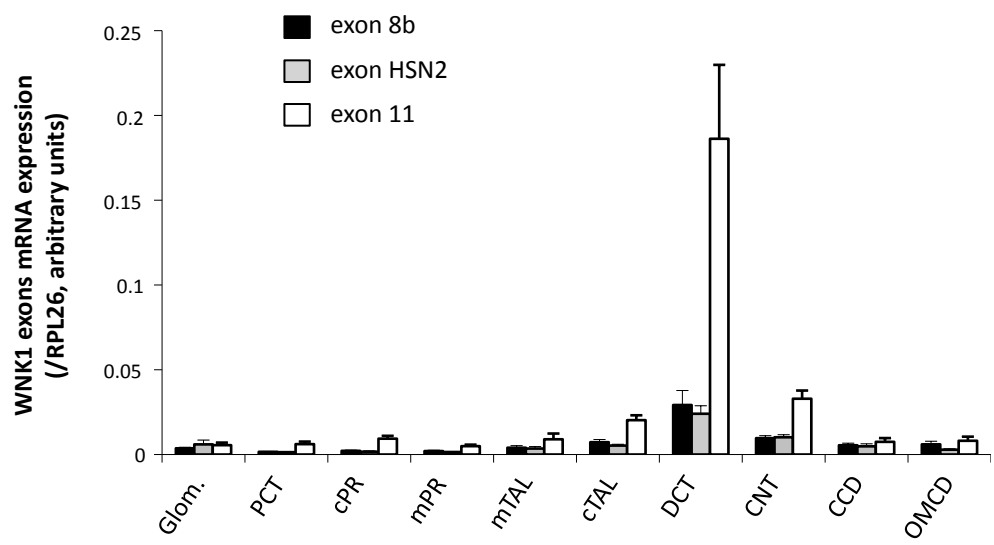

Figure S2

Supplement: Figure S2 — Pattern of expression and relative proportions of the splice variants of region 8–10 in mice. (A) Schematic representation of the different splicing events that can occur in this region. (B) Relative quantities of these variants in a panel of mouse tissues, expressed as a percentage of total WNK1 expression. Cereb.: Cerebellum; Sp. cord+DRG: Spinal cord+Dorsal Root Ganglia; Sk. muscle: Skeletal muscle. Unlike the human situation, exon 9 is always included in the mouse WNK1 mRNA, which could also contain an additional exon, 8b. (C) Expression level of exons 8b, HSN2 and 11 in microdissected mouse nephron segments, relative to RPL26. Exon 11 was included to highlight the low expression of exons 8 h and HSN2, compared to exon 12, described in Figure S3C. Datas are means ± s.e.m from 6 mice. (PDF) [file pone.0037751.s002.pdf]

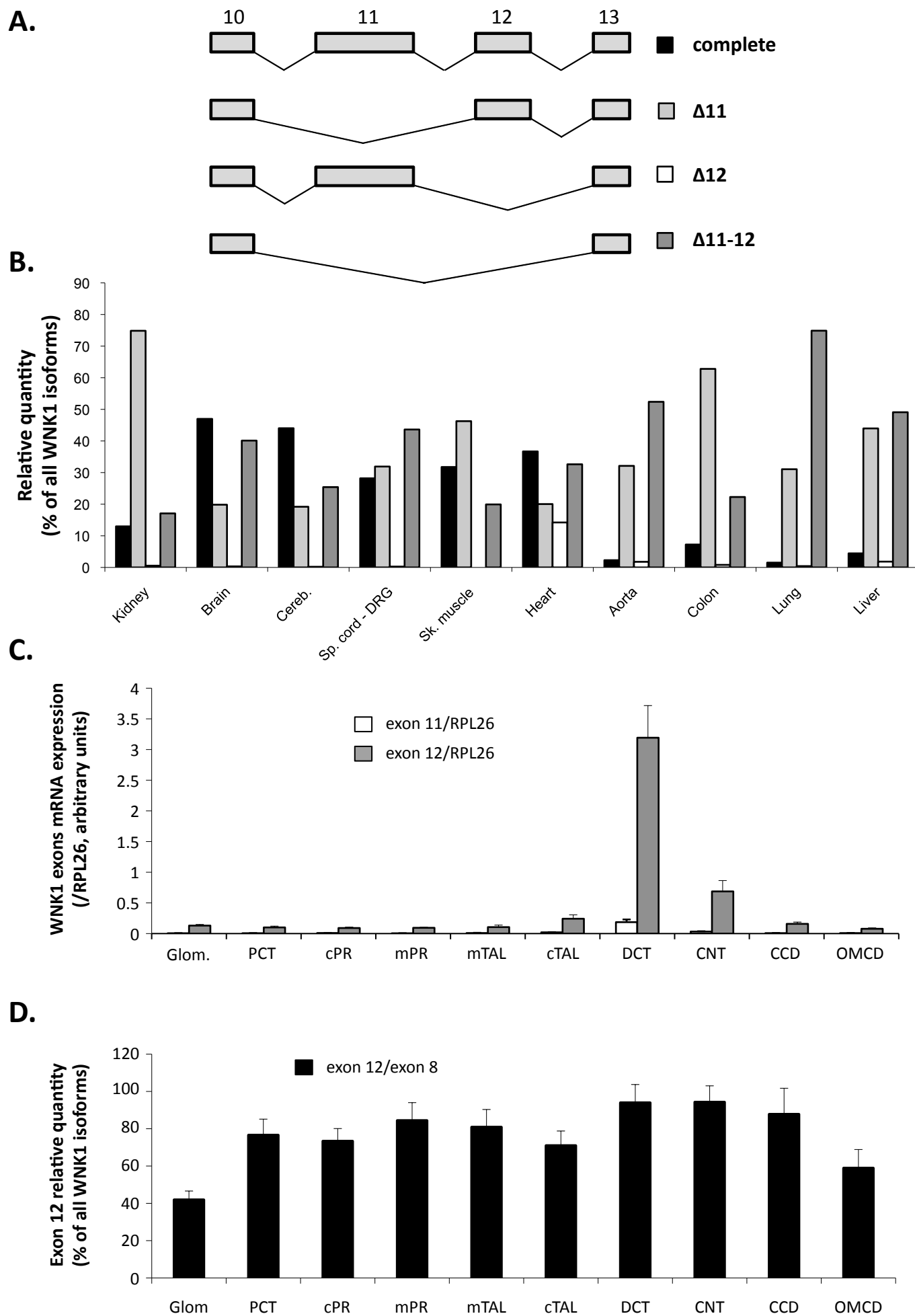

**Figure S3**

Supplement: Figure S3 — Pattern of expression and relative proportions of the splice variants of region 10–13 in mice. (A) Schematic representation of the different splicing events that can occur in this region. (B) Relative quantities of these variants in a panel of mouse tissues, expressed as a percentage of total WNK1 expression. (C) Expression level of exons 11 and 12 in microdissected mouse nephron segments, relative to RPL26. Datas are means ± s.e.m from 6 mice. (D) Relative quantity of WNK1 transcripts containing exon 12 in microdissected mouse nephron segments, calculated as the ratio of exon 12 over exon 8. (PDF) [file pone.0037751.s003.pdf]

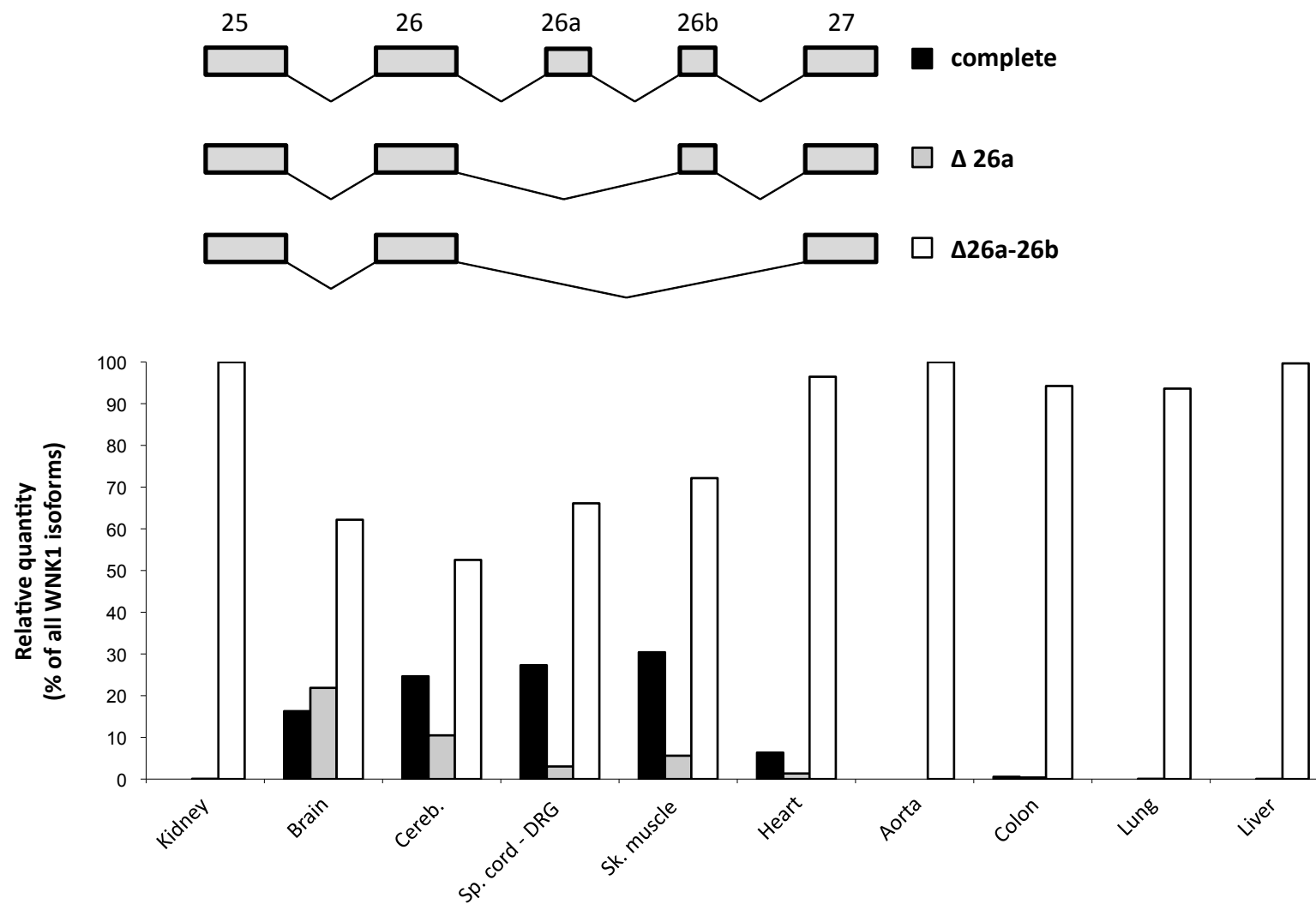

**Figure S4**

Supplement: Figure S4 — Pattern of expression and relative proportions of the splice variants of region 25–27 in mice. Upper panel: schematic representation of the different splicing events that can occur in this region. Lower panel: relative quantities of these variants in a panel of mouse tissues, expressed as a percentage of total WNK1 expression. Unlike the human situation, exon 26 is never spliced out of the mouse WNK1 mRNA. (PDF) [file pone.0037751.s004.pdf]

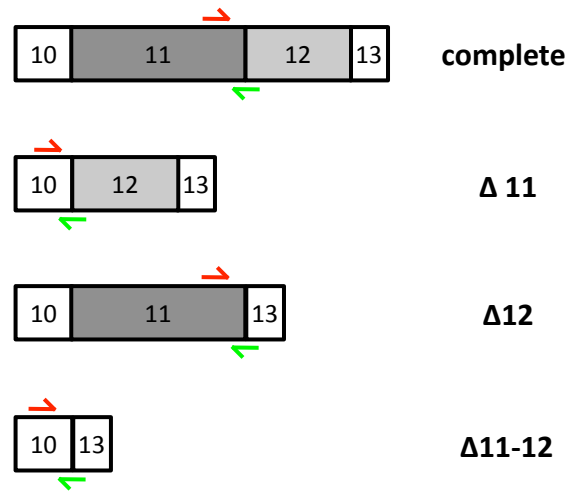

**Figure S5**

Supplement: Figure S5 — Schematic representation of the localization of the primers used to determine the relative quantity of the WNK1 isoforms. The example of region 10–13 is shown. One of the primers within one pair was designed such that it overlaps exons that are contiguous only in the isoform of interest. For example, to study the expression of the “Δ11” isoform, the forward primer recognises a sequence located in exon 10 while the reverse primer overlaps exons 10 and 12. (PDF) [file pone.0037751.s005.pdf]
