# Supplementary material for: A New Methodology for Quantification of Alternatively Spliced Exons Reveals a Highly Tissue-Specific Expression Pattern of WNK1 Isoforms
Source: PLoS One. 2012 May 31;7(5):e37751. doi: 10.1371/journal.pone.0037751 (PMC3365125; doi:10.1371/journal.pone.0037751)
Supplement: Table S1 — Relative quantities of WNK1 isoforms in a panel of human tissues. (PDF) [file pone.0037751.s006.pdf]

	Isoform	Primers	Tissue										
			**Kidney**	**Brain**	**Cereb.**	**Sp cord**	**DRG**	**Sk. m.**	**Heart**	**Aorta**	**Colon**	**Lung**	**Liver**
**Region 8-10**	**complete**	HSN2-9	8.7	11.9	8.2	3.4	56.8	0.8	0.4	8.2	2.5	3.0	1.0
	**ΔHSN2**	8-9	35.8	43.5	34.5	34.0	19.2	44.6	71.6	39.3	55.9	38.2	49.5
	**ΔHSN2-9**	8-10	63.7	52.5	47.8	46.8	28.4	49.4	38.2	54.5	40.5	55.4	52.3
	sum		***108.2***	***107.8***	***90.5***	***84.3***	***104.4***	***94.9***	***110.2***	***102.0***	***98.9***	***96.7***	***102.8***
**Region 10-13**	**complete**	11-12	16.2	19.8	25.8	4.4	7.0	53.3	73.3	12.4	12.9	5.7	7.2
	**Δ11**	10-12	66.9	20.1	35.9	20.4	49.1	8.8	3.8	9.6	19.7	20.8	25.8
	**Δ12**	11-13	6.1	7.3	9.8	1.7	2.3	17.9	29.9	6.4	5.6	2.2	3.0
	**Δ11-12**	10-13	24.1	41.7	29.4	64.7	57.6	40.5	17.8	69.2	35.6	55.2	53.1
	sum		***113.3***	***88.9***	***101.0***	***91.1***	***115.9***	***120.5***	***124.8***	***97.6***	***73.8***	***84.0***	***89.1***
**Region 25-27**	**complete**	26a-26b	0.0	13.5	33.1	7.1	43.3	18.7	1.7	0.1	0.6	0.0	0.0
	**Δ26a**	26-26b	0.2	8.0	5.6	2.8	0.2	0.9	0.2	0.4	0.1	0.1	0.1
	**Δ26a-26b**	26-27	93.1	81.7	47.4	70.5	48.5	84.6	84.2	99.9	81.4	95.9	98.4
	**Δ26-26a-26b**	25-27	10.0	7.3	8.9	4.7	8.9	4.5	5.1	5.0	5.5	6.1	4.3
	sum		***103.3***	***110.5***	***94.9***	***85.2***	***100.9***	***108.7***	***91.1***	***105.4***	***87.5***	***102.0***	***102.8***

Table S1. Relative quantities of WNK1 isoforms in a panel of human tissues.
