# Supplementary material for: A New Methodology for Quantification of Alternatively Spliced Exons Reveals a Highly Tissue-Specific Expression Pattern of WNK1 Isoforms
Source: PLoS One. 2012 May 31;7(5):e37751. doi: 10.1371/journal.pone.0037751 (PMC3365125; doi:10.1371/journal.pone.0037751)
Supplement: Table S2 — Relative quantities of WNK1 isoforms in a panel of mouse tissues. * Spinal cord samples also contain the Dorsal Root Ganglia. (PDF) [file pone.0037751.s007.pdf]

	Isoforms	Primers	Tissue									
			**Kidney**	**Brain**	**Cereb**	**Sp.cord***	**Sk. m.**	**Heart**	**Aorta**	**Colon**	**Lung**	**Liver**
**Region 8-9**	complete	8-8b	0.1	5.9	8.7	39.5	0.3	0.1	12.0	0.3	0.1	0.2
	Δ8b	8-HSN2	0.4	16.0	9.5	18.0	0.5	0.1	0.0	0.9	0.4	0.1
	Δ8b-HSN2	8-9	102.1	78.8	75.7	49.2	108.2	96.5	81.5	89.4	94.9	98.4
	sum		***102.7***	***100.7***	***93.9***	***106.7***	***108.9***	***96.7***	***93.5***	***90.6***	***95.4***	***98.6***
**Region 10-13**	complete	11-12	13.0	47.0	44.0	28.2	31.8	36.7	2.3	7.2	1.5	4.5
	Δ11	10-12	74.9	19.8	19.2	31.9	46.3	20.0	32.1	62.8	31.1	44.0
	Δ12	11-13	0.6	0.3	0.2	0.3	0.0	14.2	1.8	0.8	0.4	1.8
	Δ11-12	10-13	17.1	40.1	25.4	43.6	19.9	32.6	52.4	22.3	74.9	49.1
	sum		***105.5***	***107.3***	***88.8***	***104.0***	***98.0***	***103.6***	***88.6***	***93.1***	***107.9***	***99.4***
**Region 26-27**	complete	26a-26b	0.0	16.3	24.7	27.3	30.4	6.4	0.0	0.6	0.0	0.0
	Δ26a	26-26b	0.1	21.9	10.5	3.0	5.6	1.3	0.0	0.4	0.1	0.1
	Δ26a-26b	26-27	99.9	62.2	52.5	66.1	72.2	96.5	99.9	94.2	93.6	99.6
	sum		***100.0***	***100.3***	***87.7***	***96.4***	***108.2***	***104.2***	***99.9***	***95.2***	***93.7***	***99.7***

Table S2. Relative quantities of WNK1 isoforms in a panel of mouse tissues. * Spinal cord samples also contain the Dorsal Root Ganglia.
