# Supplementary material for: A New Methodology for Quantification of Alternatively Spliced Exons Reveals a Highly Tissue-Specific Expression Pattern of WNK1 Isoforms
Source: PLoS One. 2012 May 31;7(5):e37751. doi: 10.1371/journal.pone.0037751 (PMC3365125; doi:10.1371/journal.pone.0037751)
Supplement: Table S3 — Relative quantities of L-WNK1 and KS-WNK1 in microdissected mouse nephron segments. [L-WNK1]Q and [KS-WNK1]Q are the values of the ratio of each transcript over exon 8, given by the 2−ΔCT formula. [L-WNK1] and [KS-WNK1] are the “true” expression ratio. α and β are the calculated correction factors. The ratios given here, being expressed over exon 8, represent the proportion of L-or KS-WNK1 amongst all WNK1 transcripts. Glom: Glomerulus - PCT: Proximal Convoluted Tubule - cPR: cortical Pars Recta - mPR: medullary Pars Recta - mTAL: medullary Thick Ascending Limb of Henle's loop - cTAL: cortical Thick Ascending Limb of Henle's loop - DCT: Distal Convoluted Tubule - CNT: Connecting tubule - CCD: cortical Collecting Duct - OMCD: Outer Medullary Collecting Duct. (PDF) [file pone.0037751.s008.pdf]

Isoform	Primers	Nephron segment									
		**glom**	**PCT**	**cPR**	**mPR**	**mTAL**	**cTAL**	**DCT**	**CNT**	**CCD**	**OMCD**
**$[L\text{-}WNK1]_Q$**	ex 2-3	*77.22*	*62.59*	*78.49*	*78.09*	*49.62*	*28.15*	*0.98*	*10.30*	*54.87*	*72.77*
**α**						1.26					
**[L-WNK1]**		97.29	78.87	98.90	98.39	62.52	35.47	1.24	12.98	69.14	91.70
**$[KS\text{-}WNK1]_Q$**	ex 4a-5	*0.60*	*4.66*	*2.25*	*1.18*	*16.22*	*26.75*	*41.15*	*33.53*	*15.00*	*3.70*
**β**						2.4					
**[KS-WNK1]**		1.43	11.19	5.41	2.83	38.93	64.19	98.77	80.48	36.00	8.88
**[L-WNK1] + [KS-WNK1]**		**98.72**	**90.06**	**104.30**	**101.22**	**101.45**	**99.66**	**100.01**	**93.45**	**105.13**	**100.57**

Table S3. Relative quantities of L-WNK1 and KS-WNK1 in microdissected mouse nephron segments. $[L\text{-}WNK1]_Q$ and $[KS\text{-}WNK1]_Q$ are the values of the ratio of each transcript over exon 8, given by the $2^{-\Delta CT}$ formula. [L-WNK1] and [KS-WNK1] are the "true" expression ratio. α and β are the calculated correction factors. The ratios given here, being expressed over exon 8, represent the proportion of L-or KS-WNK1 amongst all WNK1 transcripts. Glom: Glomerulus - PCT: Proximal Convoluted Tubule - cPR: cortical Pars Recta - mPR: medullary Pars Recta - mTAL: medullary Thick Ascending Limb of Henle's loop - cTAL: cortical Thick Ascending Limb of Henle's loop - DCT: Distal Convoluted Tubule - CNT: Connecting tubule - CCD: cortical Collecting Duct - OMCD : Outer Medullary Collecting Duct
